# Semi-Reliability Probability Damage Assessment of GFRP Bars Embedded in Steam-Curing Concrete Beams Based on the Multiple Factors Related Moisture Absorption Model

**DOI:** 10.3390/polym13244409

**Published:** 2021-12-16

**Authors:** Kai Zhang, Wenrui Yang, Huiying Li, Zhiyi Tang, Weiwei Wu, Jiao Yuan, Zhongmin Feng

**Affiliations:** 1 Department of Road and Materials, Jiangxi Transportation Institute, Nanchang 330012, China; kaizhang@whut.edu.cn; 2 School of Civil and Architectural Engineering, East China University of Technology, Nanchang 330012, China; lhying13@163.com (H.L.); tzhiyi7@163.com (Z.T.); m15270028010_1@163.com (J.Y.); m906836738@163.com (Z.F.); 3 School of Transportation and Logistics Engineering, Wuhan University of Technology, Wuhan 430063, China; www92@whut.edu.cn; 4 Hubei Province Highway Engineering Research Center, Wuhan 430063, China

**Keywords:** steam-curing concrete, GFRP bar, moisture absorption, damage assessment, semi-reliability

## Abstract

GFRP bars will be damaged due to a series of irreversible hygroscopic chemical reactions under humid and hot curing environmental conditions. The multiple factors related to the moisture absorption model were established through the moisture absorption test of GFRP bars embedded in steam-curing concrete, which considered different curing temperatures, different thicknesses of the protective layer, and different diameters of GFRP bars. Semi-reliability probability damage assessment of GFRP bars embedded in steam-curing concrete was described by introducing the reliability and stochastic theory. Subsequently, the tensile test of GFRP bars was carried out to verify the feasibility of the damage assessment. The results showed that the moisture absorption curves of GFRP bars were basically in line with Fick’s law. In addition, the influences of the curing temperature, the thickness of the protective layer, and the diameter on moisture absorption performance were presented. The semi-reliability probability damage assessment model of GFRP bars embedded in steam-curing concrete beams adequately considered the multiple factors related to moisture absorption and the uncertainty and randomness of the influencing factors during the process of moisture absorption.

## 1. Introduction

Hot and humid steam-curing accelerates cement hydration, accompanied by the development of the compressive strength and decrease of its permeability in hours [1]. Meanwhile, it significantly impacts the mechanical and durability properties of reinforced bars embedded in steam-curing concrete beams due to the rise of the temperature inside the concrete during the curing process [2]. According to statistics, the poor durability of steam-curing precast concrete components is the main issue in the service process. Generally, the actual service life of 20 years is much less than the design life of 50 years [3,4,5]. FRP bars have the characteristics of light weight, high strength and strong corrosion resistance. Among them, glass fiber reinforced polymer (GFRP) bars have become the focus of engineering circles because of their relatively low price advantage [6]. The use of FRP bars in concrete structures subjected to harsh environments generates considerable potential for extending the service life of these structures and lowering their overall life cycle cost [7]. In high-speed railway engineering, GFRP bars with corrosion resistance and superior cost-effectiveness are widely used in the steam-curing of precast concrete by completely or partly replacing steel bars, which can improve the durability of the steam-curing precast concrete components [8,9,10]. The difference in the tensile strength damage between GFRP bars and steel bars embedded in steam-curing concrete beams has been identified. With the curing temperature of 60 °C, the deterioration of the tensile strength of GFRP bars is less than 10%. However, the damage of the steel bars reaches 20% or even higher. Therefore, it can be inferred that combined with GFRP bars, the deterioration of steel bars will become more obvious in the service process [11].

Researchers explored the risk factors associated with the damage of GFRP bars, including hygrothermal effect, chemical corrosion, oxidation, and light radiation. Compared with the glass fiber in the resin phase, the resin with faster moisture absorption and higher expansibility has a pronounced effect on the moisture and heat of the composite. This effect will lead to stress failure at the debonding interface and matrix cracking damage, thereby reducing the mechanical properties of GFRP bars [12,13,14,15,16,17]. Xu Jian revealed that the moisture absorption rate of the glass fiber reinforced polymer laminates was accelerated in the hygrothermal environment. Meanwhile, the moisture absorption rate calculated by the equivalent method could be used to characterize the three-dimensional moisture absorption process of the composites [18].

The moisture absorption process of GFRP bars is much more complex. Although Fick’s law is no longer applicable to moisture diffusion in heterogeneous materials, the law of distribution in GFRP bars is still relevant. Xue et al. tested the tensile properties of glass fiber plastic (GFRP) bars with stress levels of 0%, 25%, and 45%, respectively, in an alkali environment at 60 °C. The research showed that the diffusion process of OH^−^ ions in GFRP Bars conforms to Fick’s law, and a regression model of tensile strength of stressed GFRP Bars in an alkali environment was proposed [19]. Li et al. used the water absorption of GFRP to calculate the erosion diffusion coefficient of the solution medium, and an FRP point source erosion depth model based on Fick’s law was proposed [20]. Katsuki et al. carried out an accelerated test on FRP reinforcement with an alkaline solution. The deterioration of tensile strength of GFRP rods was simulated quantitatively using Fick’s first law [21]. The effective water diffusivity of the GFRP bar depends on the volume ratio, saturated water content, and other factors, which are mainly used to characterize the comprehensive characteristics of each component. Especially for the GFRP bars with damage, the matrix crack, the debonding interface, and the interlayer crack will undoubtedly increase the effective moisture diffusivity of the material [22,23,24,25]. Therefore, this study on the moisture absorption performance of GFRP bars has a special relevance to its damage assessment and the service life, especially in the hygrothermal environment that causes the damage of GFRP bars. However, up to now, far too little attention has been paid to the influence of the moisture absorption performance on the deterioration of GFRP bars embedded in steam-curing concrete beams during the curing process.

Specifically, this paper aims to propose the damage assessment of GFRP bars embedded in steam-curing concrete beams based on the multiple factors related to the moisture absorption model—the curing temperature, the protective layer’s thickness, and the diameter of the GFRP bars. Finally, the uncertainty and randomness of damage factors during the process of moisture absorption are comprehensively considered.

## 2. Experimental

### 2.1. Materials and Specimen Preparation

Sand-coated GFRP bars made up of unidirectional roving of 70% E-glass and 30% epoxy vinyl ester resin used in the study, were received from the Fenghui Composite Materials Co., Ltd., Nanjing, China. The selected bars with different diameters of 10, 16, 19, and 22 mm were tested to study the influence of diameter on the moisture absorption property of GFRP bars embedded in concrete beams. The specimens used in the experiments included GFRP bars embedded in concrete beams and the bare GFRP bars.

**Figure 1:**
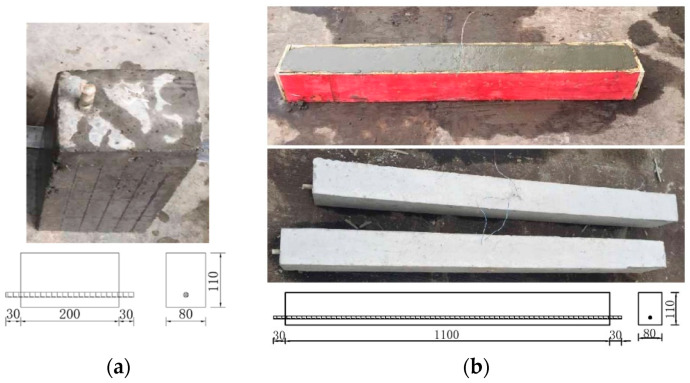
The preparation of the research sample. (**a**) specimen sizes (200 mm × 110 mm × 80 mm), (**b**) specimen sizes (1100 mm × 110 mm × 80 mm).

Following typical concrete mix design procedures, all concrete beams were cast with 75% Type I Portland cement (P.O 42.5) and 25% Class F fly ash, and the water-to-cement ratio was 0.59, where the percentages were expressed by weight. The GFRP reinforced concrete beams specimens were rectangular plain concrete beams with two different specimen sizes (200 mm × 110 mm × 80 mm, 1100 mm × 110 mm × 80 mm), with a single longitudinal GFRP bar centered at 15, 20, 25, and 35 mm from the bottom of the section. The preparation of the research sample is shown in Figure 1.

### 2.2. Curing Systems

**Figure 2:**
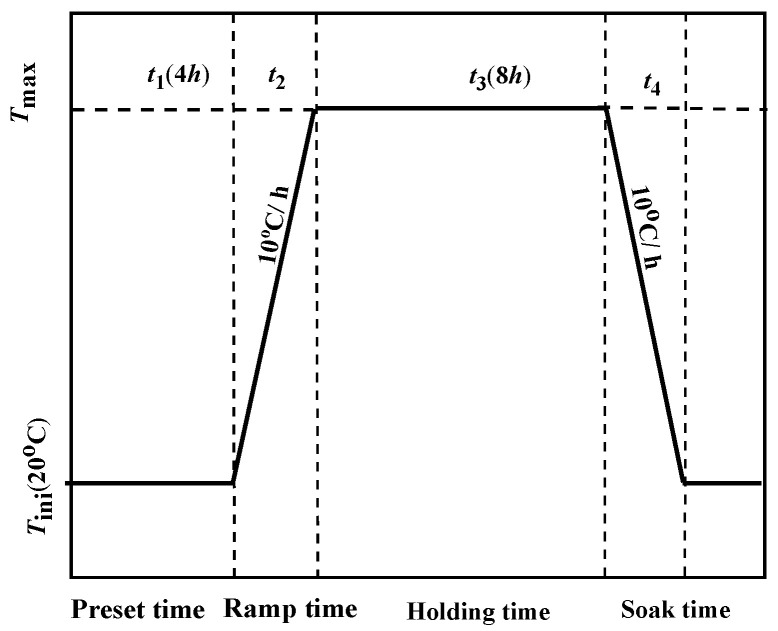
Steam-curing System.

In the curing process, the hydration of concrete is related to the curing temperature, heating rate, and cooling rate [26,27]. In this paper, the steam curing system is carried out according to the relevant provisions in the standard for construction quality acceptance of highspeed railway bridge and culvert engineering [28]. During all the steps, both heating and cooling rates were controlled and fixed in all cases at 10 °C/h to analyze the influence of the curing temperature on the moisture absorption property of GFRP bars embedded in concrete beams. Once the specimens were cast, they were kept inside the environmental chamber with a relative humidity of 90% and an initial temperature *T*_ini_ (20 °C) for a preset time *t*_1_ (4 h). Then, the curing temperature was raised to the target temperature *T*_max_ (20, 60 and 80 °C) at a rate of 10 °C/h and lasted for 8 h (Figure 2). In addition, it was defined as the standard-curing when the curing temperature had been kept at the initial temperature *T*_ini_ (20 °C).

### 2.3. Moisture Absorption Tests

Moisture absorption analysis was presented based on the accelerated test method in a constant temperature water bath to absorb moisture in their internal structures. A moisture uptake profile depicts the relationship between time and the amounts of moisture vapor-material exchanges at a given temperature (60 °C) and relative humidity (100%). The dry sample mass calculates the moisture absorption rate initially and the wet sample mass at a given time with 1, 25, 37, 92 and 182 d (Equation (1)). All GFRP bars were placed in the oven for 24 h before the moisture absorption rate test to ensure their quality without any change in the case. The accuracy of the electronic balance used in the test was 0.01 g.

(1) $$M=\frac{M_{1}-M_{0}}{M_{0}}\times 100\%$$

where *M*_1_ is the wet sample mass after time *t*; *M*_0_ is the dry sample mass at the initial time, and *M* is the moisture absorption rate.

### 2.4. Tensile Strength Damage Test

For testing, all tensile specimens were prepared by anchoring two ends of the bars in steel plugs filled with epoxy resin. The free length between the two steel plugs was about 300 mm to ensure that the anchor bonding strength was higher than the tensile stress according to the guidelines as specified in ACI 440.3R-04 [29]. The test was carried out with a universal testing machine (SHT4106-G, Jinan MTS Test Technology Co., Ltd. Jinan, China), and an extensometer of 50 mm gauge length was mounted with clips at the center of the test specimens according to ACI 440.3R-04. The applied load was recorded during the test with a data-acquisition system monitored by a computer. The related parameters of the specimens are shown in Table 1.

### 2.5. Scanning Electron Microscope (SEM) Test

**Figure 3:**
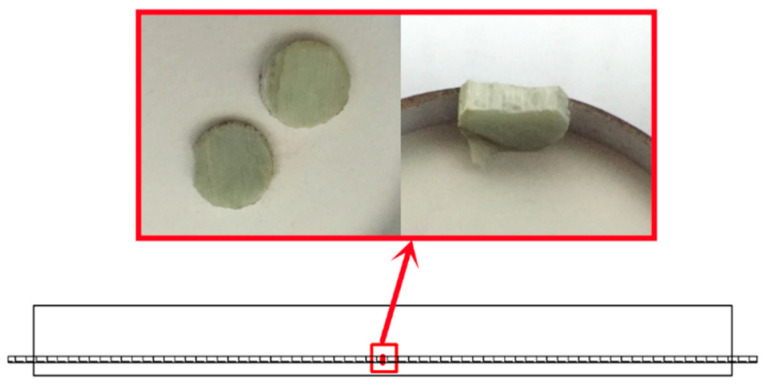
The scanning position and sample of the scanning electron microscope.

The microstructure changes of the GFRP bars with different diameters under different temperature curing environments were analyzed by a Nova nanosem450 field emission scanning electron microscope produced by FEI Company, Hillsborough, OR, USA. The sample was taken from the inside of the impermeable test block. The size of the sample is a round cake with diameters of 10 and 16 mm. During sampling, the damage to the observation surface was avoided. The scanning position and sample of the scanning electron microscope are shown in Figure 3.

## 3. Results and Discussion

### 3.1. SEM Microstructure Analysis

**Figure 4:**
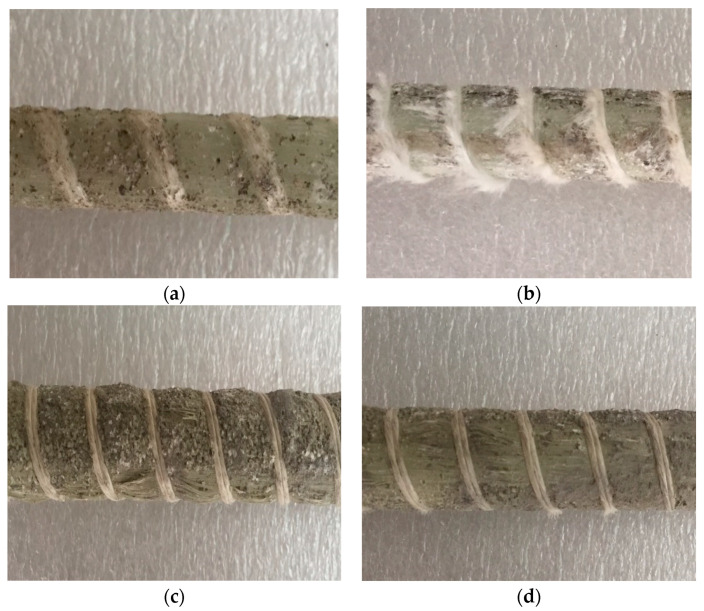
Apparent morphology of GFRP bars embedded in concrete. (**a**) GFRP bars with 10 mm diameter embedded in standard curing concrete, (**b**) GFRP bars with 10 mm diameter embedded in steam curing concrete, (**c**) GFRP bars with 16 mm diameter embedded in steam curing concrete, and (**d**) GFRP bars with 22 mm diameter embedded in steam curing concrete.

Under different conditions, with different surface damage degrees, the GFRP bars are embedded in concrete. GFRP damages such as pitting corrosion, loose glass fiber bundle, or surface sandblasting were tested. Figure 4a,b shows that the glass fiber bundle damage of GFRP bars embedded in concrete with a curing temperature of 60 °C is more obvious than that of 20 °C. Figure 4c,d shows that the glass fiber bundles on the surface of GFRP bars with larger diameter do not have any looseness, and the damage decreases with increasing diameter. It will directly affect the resin moisture absorption performance of GFRP bars embedded in concrete with different curing temperatures and different diameters.

**Figure 5:**
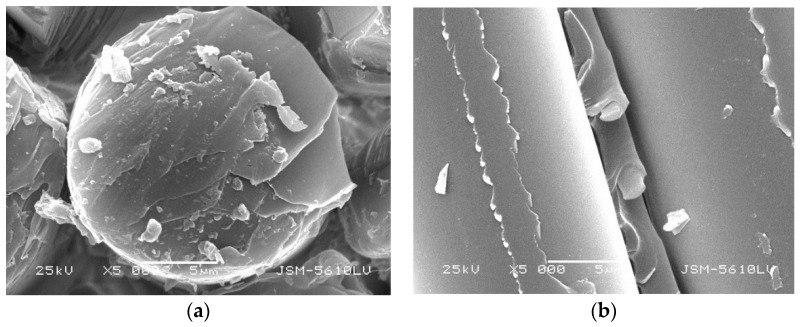
Microstructure morphology of GFRP bare bars with 10 mm diameter in standard curing. (**a**) cross section, (**b**) vertical section.

**Figure 6:**
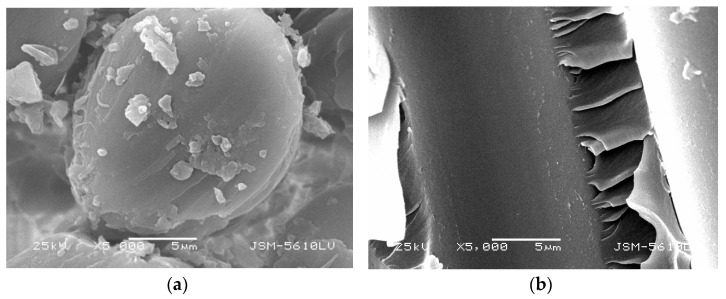
Microstructure morphology of GFRP bars with 10 mm diameter embedded in standard curing concrete. (**a**) cross section, (**b**) vertical section.

It can be seen from Figure 5 and Figure 6 that the cross-section of GFRP bars embedded in concrete is loose under the standard curing temperature of 20 °C, and there are scattered resin fragments in the vertical section. There are also some scattered resin fragments of the GFRP bare bar under the standard curing temperature of 20 °C. However, the cross-sectional and the vertical-sectional fibers are relatively smooth, and there are no pit corrosion points. It can be seen that the microstructure of GFRP bare bars does not change in the standard curing environment, but GFRP bars will be damaged in an alkaline concrete environment.

**Figure 7:**
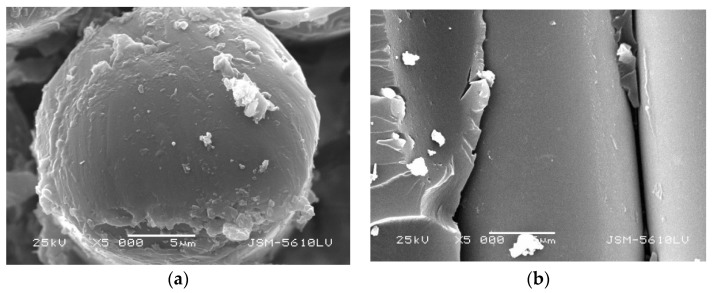
Microstructure morphology of GFRP bars with 10 mm diameter in steam curing. (**a**) cross section, (**b**) vertical section.

**Figure 8:**
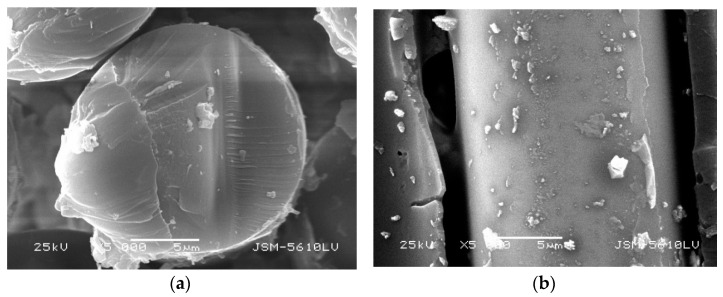
Microstructure morphology of GFRP bars with 10 mm diameter embedded in steam curing concrete. (**a**) cross section, (**b**) vertical section.

However, the microstructure of GFRP bars embedded in concrete is not significantly different from bare bars during steam curing as seen by comparing Figure 7 with Figure 8. It shows that the concrete has an alkaline deterioration effect on GFRP bars and plays an obvious isolation and protection role of high temperature and high humidity.

**Figure 9:**
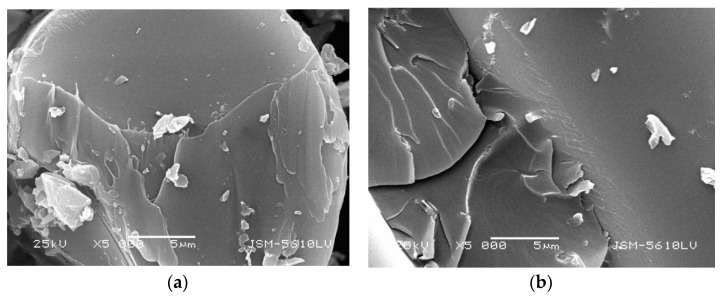
Microstructure morphology of GFRP bars with 22 mm diameter in steam curing. (**a**) cross section, (**b**) vertical section.

**Figure 10:**
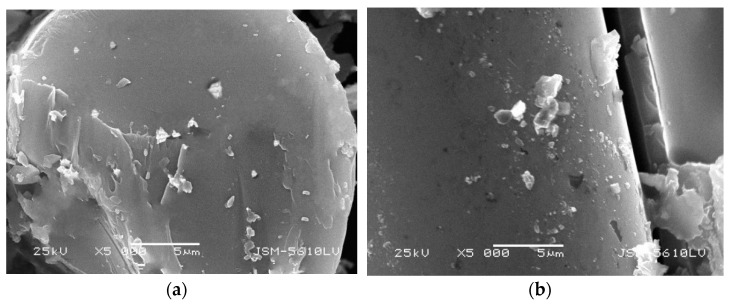
Microstructure morphology of GFRP bars with 22 mm diameter embedded in steam curing concrete. (**a**) cross section, (**b**) vertical section.

From Figure 7, Figure 8, Figure 9 and Figure 10, it can be seen that the damage of the cross and vertical sections of the GFRP bars with a diameter of 10 mm embedded in the steam curing concrete is the most obvious; the resin falls off and scatters, the fiber is loose, and pits and cracks appear. The change of microstructure morphology shows that the resin damage of GFRP bars decreases with increasing diameter. It means the damage of moisture absorption performance would decrease with increasing diameter.

**Figure 11:**
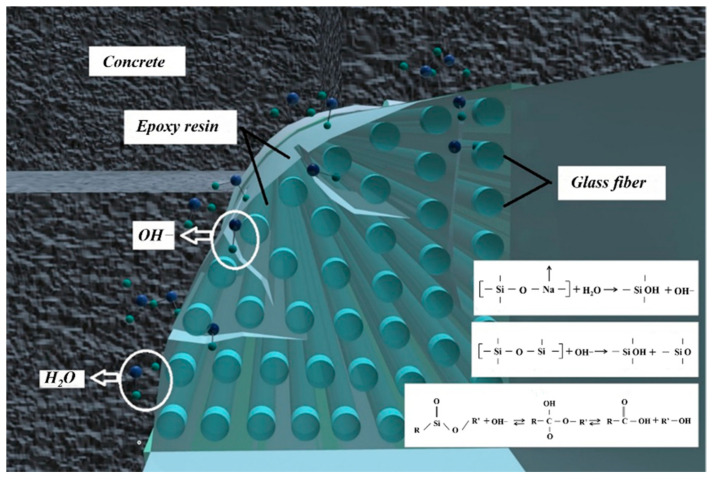
Mechanism of moisture absorption damage of GFRP bars.

The polymer matrix of GFRP bars is mainly linked by the carbon-carbon double bond and the ester groups. The moisture absorption process can be divided into the reversible physical process and the irreversible chemical process. The physical process refers to the diffusion process of free water molecules. It destroys the van der Waals force between polymer bonds in the diffusion process, which causes the expansion of the polymer matrix and the decrease of the glass transition temperature. The chemical process mainly refers to the exchange of ions among the chemical structure; the chemical exchange process will destroy the polymer matrix, which may lead to chemical hydrolysis, plastic increase, and microfracture of the polymer matrix. These phenomena will further cause the irreversible degradation of the polymer matrix. The high temperature in the curing process accelerates the chemical reaction rate of the resin matrix. It causes the irreversible degradation of the resin, resulting in the decrease of the tensile performance of the GFRP bars. Alkaline ions and other cations of the glass fiber will be exchanged with hydrogen ions in the solution to produce hydroxide solution (Figure 11).

### 3.2. Effect of Moisture Absorption Damage on Bond Strength

The surface damage of GFRP bars will directly affect the resin moisture absorption performance of GFRP bars embedded in concrete with different curing temperatures and different diameters. The change of microstructure morphology shows that the resin damage of GFRP bars decreases with increasing diameter. It means the deterioration of moisture absorption performance would decrease with increasing diameter. The maximum bond strength between steam cured concrete, and GFRP bars increases with the protective layer thickness and diameter. This shows that the hygroscopic damage of GFRP bars has a negative correlation with the bond strength between GFRP bars and concrete. That is, the greater the hygroscopic damage, the smaller the bond strength.

### 3.3. The Multi-Factor Related Moisture Absorption Rate

Figure 10 shows that the change rule of the moisture absorption rate varies with the square root of time. The moisture absorption rate was divided into two parts: the hydroxyl ion (OH^−^) quickly diffused in the internal of GFRP bars when the absorption time was short, and the diffusion absorption dynamics curve of GFRP bars could be defined as the linear phase change; however, the moisture absorption rate change was relatively smooth once the absorption time reached a certain value, which could be considered to reach the equilibrium stage. This phenomenon was consistent with the typical moisture absorption performance of FRP materials proposed by Ramirez [30,31,32].

#### 3.3.1. Different Curing Temperatures

**Figure 12:**
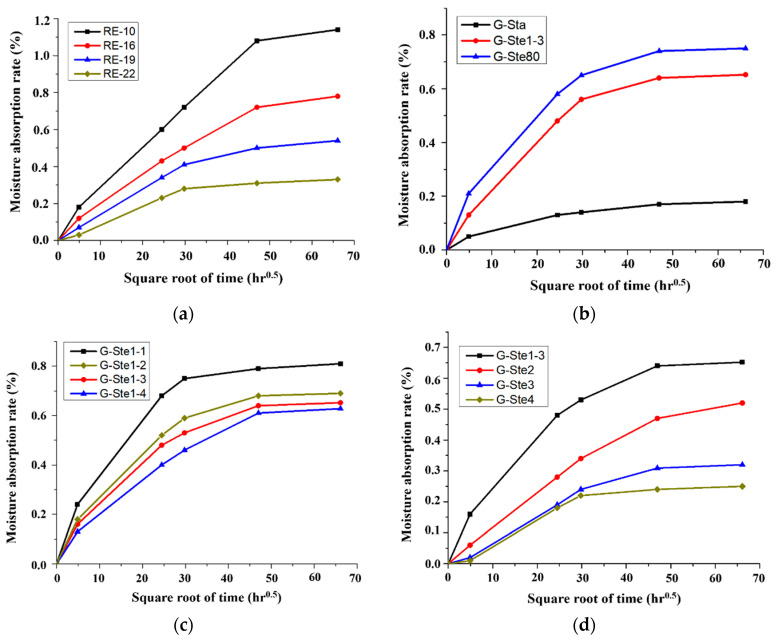
Moisture absorption rate of GFRP bars as a function of the square root of time. (**a**) GFRP bare bars, (**b**) different curing temperatures, (**c**) different thicknesses of concrete cover, (**d**) different diameters of GFRP bars.

The maximum moisture absorption rate of GFRP bare bars reached 8 times that of GFRP bars embedded in standard-curing concrete, as shown in Figure 12a,b. The maximum moisture absorption rate and the slope of the linear stage of GFRP bars embedded in steam-curing concrete beams, respectively, were 2.72 times and 3 times that of GFRP bars embedded in standard-curing concrete beams. It revealed different influences between the GFRP bare bars and GFRP bars embedded in the concrete structure. The impact of the steam-curing concrete environment on the moisture absorption rate of GFRP bars was the most obvious compared to the bare and the standard concrete environment, and the degree of influence increased with the increase of the curing temperature [2,3,33].

#### 3.3.2. Different Thicknesses of Protective Layer

It can be seen from Figure 12c that the maximum moisture absorption rate and the slope of the linear stage both decrease with increasing thickness of the protective layer. The maximum moisture absorption rates of GFRP bars embedded in steam-curing concrete beams with the protective layer of 15, 20, 25, and 35 mm were approximately 0.81%, 0.69%, and 0.628%, respectively. The slopes of the linear stage were 2.1, 1.6, 1.4, and 1.3, respectively. Therefore, the protective layer thickness of 15 mm has the most obvious effect on the maximum moisture absorption rate of the GFRP bar. The damage on the surface of steam cured concrete provides more channels for the transfer of external water and heat, resulting in the acceleration of the moisture absorption reaction of the GFRP bar.

#### 3.3.3. Different Diameters of GFRP Bars

The rate of the internal chemical reaction of different diameters will be quite different. Therefore, the diameter of GFRP bars is the main variable in studying the moisture absorption of GFRP bars. Figure 12d shows that the moisture absorption rate of GFRP bars increases with the increase in diameter. Nevertheless, the slope of the linear stage decreases with increasing diameter. The maximum moisture absorption rates of GFRP bars with a diameter of 10, 16, 19, and 22 mm embedded in steam-curing concrete beams, respectively, were 0.652%, 0.52%, 0.318%, and 0.25%, which respectively were 0.57, 0.67, 0.59 and 0.76 times that of the bare steel. The slopes of the linear stage of GFRP bars with a diameter of 10, 16, 19, and 22 mm embedded in steam-curing concrete beams were 1.5, 1.1, 0.9, and 0.8, respectively.

### 3.4. Multi-Factor Related Diffusion Coefficient D

When predicting the tensile strength of GFRP bars based on Fick’s law prediction method, it is mainly considered that the diffusion of solution medium into GFRP bars leads to the degradation of structural performance. The following basic assumptions are made for the model:

(1) Ignoring that the fiber and resin are affected by tensile force transmission in the X erosion depth area and are not eroded, the tensile properties are consistent with those before corrosion;
(2) Using OH^−^ as the only erosion ion, the FRP bars are uniformly eroded, and the time of chemical reaction between OH^−^ and fiber is ignored;
(3) OH^−^ ions only consider physical processes in the resin.

**Figure 13:**
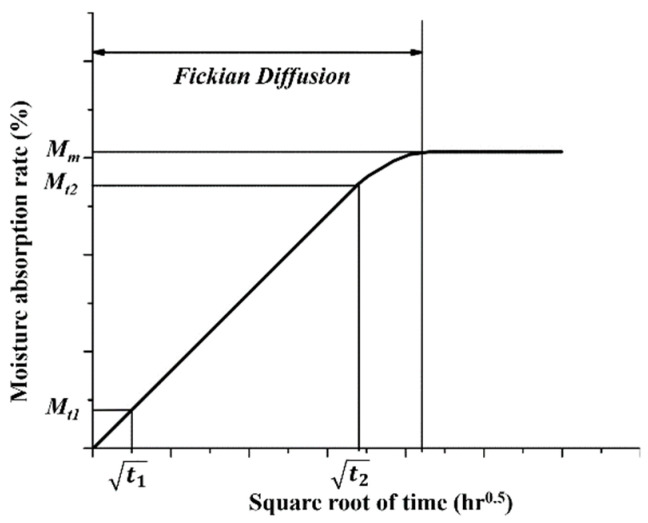
Typical absorption behavior of FRP composite.

Based on the correlation analysis of the moisture absorption rate of GFRP bars embedded in steam-curing concrete beams, it is found that the moisture absorption rate change is related to the diameter, curing temperature and the thickness of the protective layer. However, the current prediction model in Fick’s law did not consider the effect of the different diameters on the diffusion coefficient of GFRP bars, which resulted in a certain error of the tensile strength of GFRP bars between predicted and actual values. Therefore, this section will analyze the diffusion coefficient of GFRP bars for different curing temperatures, diameters, and thicknesses of the protective layer. The diffusion coefficient *D* in Fick’s law can be expressed as:

(2) $$D=\frac{\pi r^{2}}{16M_{m}^{2}}\left(\frac{M_{t_{2}}-M_{t_{1}}}{\sqrt{t_{2}}-\sqrt{t_{1}}}\right)$$

where *r* is the radius of GFRP bars; *M_m_* is the equilibrium moisture absorption rate; *M_t_*_1_ is the moisture absorption rate at the square root of time $\sqrt{t_{1}}$, and *M_t_*_2_ is the moisture absorption rate at the square root of time $\sqrt{t_{2}}$ (Figure 13).

**Figure 14:**
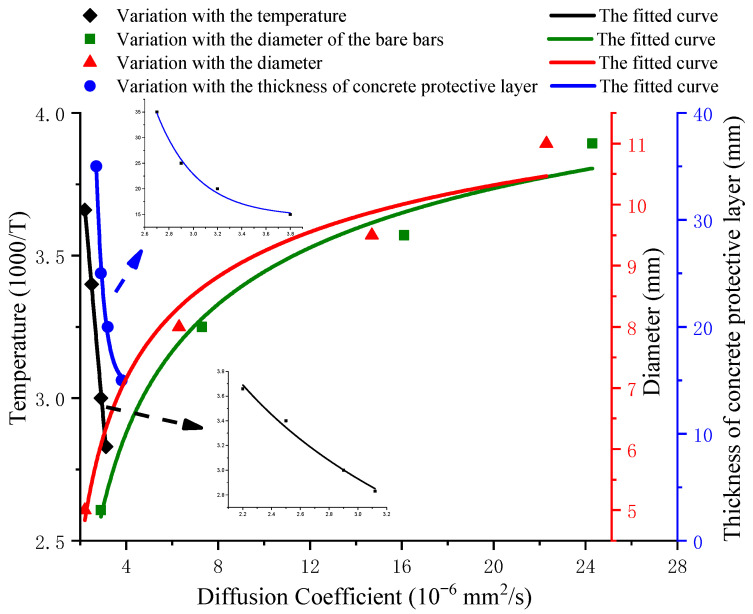
The variation of the diffusion coefficient.

Table 2 shows that the diffusion coefficient of GFRP bars embedded in standard-curing concrete beams is 1.14 times that of bare bars, mainly due to the impact of the alkaline concrete on GFRP bars. However, its influence on the standard-curing process is relatively small. The diffusion coefficient *D* will increase with the increase of curing temperature and decrease with the rise of the concrete protective layer thickness. The diffusion coefficients of GFRP bars with a diameter of 10, 16, 19 and 22 mm diameter respectively were 3.2 × 10^−6^ mm^2^/s, 7.3 × 10^−6^ mm^2^/s, 16.1 × 10^−6^ mm^2^/s and 24.3 × 10^−6^ mm^2^/s, which respectively were 1.34 times, 1.16 times, 1.10 times and 1.08 times that of bare bars. At the same time, Figure 14 shows the significant influence of diameter on the diffusion coefficient of GFRP bars. Thus, the diffusion coefficient of GFRP bars embedded in steam-curing concrete beams increased with the diameter increase. However, the influence gap between the steaming-curing concrete and bare bars decreased with the increase of the diameter in Figure 13. It is mainly caused by the comprehensive effect of the curing temperature and the thickness of the protective layer on its production.

Figure 14 shows that the variation of the diffusion coefficient of GFRP bars embedded in concrete with the curing temperature can be expressed in the form of exponential change, and the diffusion coefficient decreases with increasing the thickness of the protective layer in a power function with a negative exponent.

(3) $$D_{T}=1.01\times 10^{-5}e^{\left(\frac{-414.55}{T}\right)}$$

(4) $$D_{c}=1.16\times 10^{-5}c^{-0.4275}$$

where *T* is the maintenance of absolute temperature (K); *C* is the thickness of the protective layer; *D_T_* is the diffusion coefficient corresponding to the maintenance temperature *T*, and *D_c_* is the diffusion coefficient corresponding to the thickness of the protective layer *C*.

**Figure 15:**
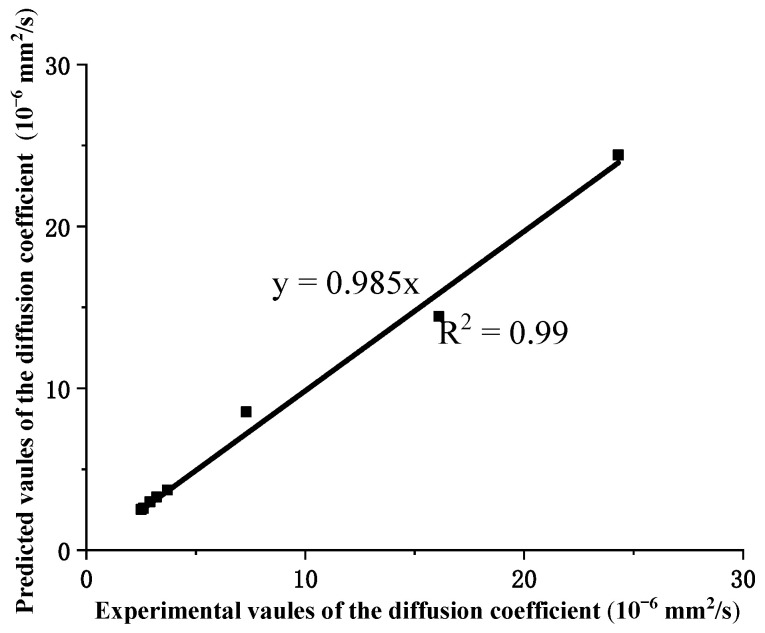
Compared with the predicted values and experimental values.

Based on the experimental results of data fitting, the data integration model was established by simultaneously considering the influence of the different curing temperatures, different diameters, and different thicknesses of the protective layer on the diffusion coefficient of the GFRP bars:

(5) $$D=\alpha e^{\left(\frac{-414.55}{T}\right)}e^{\left(0.37r\right)}c^{-0.4136}$$

where *α* is the experimental fitting value of 7.13 × 10^−6^; and *D* is the diffusion coefficient of GFRP bars. It is shown that the fitted diffusion coefficient model is credible by comparing the fitting values and experimental results (Figure 15).

Equation (5) shows an inverse relationship between the diffusion coefficient of GFRP bars and the curing temperature, namely, the diffusion coefficient increases with increasing of the curing temperature. It will enable a faster internal rate of transfer of the external solution into the GFRP bars and will accelerate the degradation performance of GFRP bars. It is revealed that the damage of GFRP bars is more obvious with the increasing steam-curing temperature.

## 4. Semi-Reliability Probability Damage Assessment

### 4.1. Semi-Reliability Probability Model

Based on certain assumptions and uncertainties, some errors were presented in the prediction equations of Fick’s law [34]. In this section, a semi-reliability probability model with a damage coefficient was proposed based on Fick’s law. In the uncertainty and randomness of damage factors, the correlation of damage factors is used to provide damage coefficients with different reliability guarantees. The model considered the time-dependent bond strength between the glass fiber and the matrix with time and introduced the multiple factors related diffusion coefficient of GFRP bars with different curing temperatures, different thicknesses of the protective layer, and different diameters. It is assumed that there is a functional relationship between the corrosion depth and the diffusion coefficient of GFRP bars. The basic principle of Fick’s law prediction model is based on the factors that may affect the moisture absorption of GFRP bars in a steam curing environment. The prediction model calculates the relationship between the initial tensile strength and the predicted tensile strength at the time point.

(6) $$\sigma_{t}={\left(1-\frac{\sqrt{2DCt}}{R_{0}}\right)}^{2}\sigma_{0}$$

where *σ*_0_ and *σ_t_* are the tensile strengths of the GFRP bars before and after curing, respectively; *R*_0_ is the radius of GFRP bars; *D* is the curing temperature; *T_c_* is the diffusion coefficient (mm^2^/s); *C* is the internal alkaline solution concentration (mol/L), and *t* is the curing time (s).

In order to adapt to the uncertainty of *σ*_0_ and *σ_t_*, and fit for the semi-reliability probability theory research, the model presented in Equation (6) can be modified by introducing the related random variables of the damage rate *β* and the parameter of the curing time *t*,

(7) $$\sigma_{t}\left(M,N\right)=\left[\left(1+S_{0}\varepsilon_{0}\right)-\beta \left(\frac{D_{T_{c}}\cdot t}{R_{0}{}^{2}}\right)\left(1+S\varepsilon \right)\right]\mu_{\sigma_{0}}$$

where *M* = (*R*_0_, *D*) as the basic vector (i.e., the radius of the GFRP bar and the polymer diffusion coefficient at the time of *t* = 0); *ε*_0_ and *ε* are random variables with the independent zero mean and the unit variance, respectively; *S*_0_ and *S* are respectively the standard deviations of *ε*_0_ and *ε*; *S*_0_*ε*_0_ is the error term for the variable *σ*_0_ with the mean value of $\mu_{\sigma_{0}}$; *Sε* is the error term of the degenerate term $\beta {\left(D_{T_{c}}\cdot t/R_{0}{}^{2}\right)}^{\alpha }$, and $N=\left(\beta ,\alpha ,S_{0},S\right)$ is an unknown empirical model parameter vector to adapt to the test data. The above model has two hypotheses (which can be proven effective): *S*_0_, *S* are independent of *R*_0_; *ε*_0_ and *ε* have a normal distribution.

To simplify the prediction model, the moisture damage factors with *λ* and *γ* are introduced, in which *λ* is related to different thicknesses of the protective layer and different diameters of the diffusion coefficient, and *γ* indicates the moisture absorption rate of GFRP bars varies with time. Finally, the modified prediction model of the tensile strength of GFRP bars embedded in steam-curing concrete beams is obtained by simplified Equation (8):

(8) $$\sigma_{t}\left(M,N\right)=\left[\left(1+S\varepsilon_{0}\right)-\lambda t^{\gamma }\left(1+S\varepsilon_{0}\right)\right]\sigma_{0}$$

The key to the damage evaluation of GFRP reinforcement with tensile strength as the characteristic value by using the above prediction model formula is to determine the unknown parameters in the formula. The undetermined parameters are mainly *β*, *α*, *S*_0_ and *S*, because these parameters are used to fit the mathematical model with the empirical data, they do not have specific physical significance in the determination process. This paper aims to determine the above parameters by a statistical method based on a small amount of empirical data of tensile strength change of GFRP reinforcement. According to the statistical principle, in the above formula *ε* and *ε*_0_ follow the normal distribution, so the error term *s*·*ε* obeys (0, *S*^2^) distribution; *s*_0_·*ε*_0_ obeys the (0, *S*_0_^2^) distribution; $S\cdot \varepsilon \cdot \beta {\left(D_{T_{c}}\cdot t/R_{0}{}^{2}\right)}^{\alpha }$ obeys the (0, *S*^2^, $\beta^{2}{\left(\frac{D_{T_{c}}\cdot t}{R_{0}{}^{2}}\right)}^{2\alpha }$) distribution.

According to the test data and Equation (5), the moisture damage factors, *λ* and *γ*, can be determined. With the steam-curing temperature of 60 °C, the value *γ* is 1.69 and *λ* is defined as the parameter related to the thickness of protective layer and the diameter of the GFRP bars:

(9) $$\lambda =2.79\times 10^{-11}e^{0.175r}c^{-0.2135}/r^{2}$$

### 4.2. Damage Assessment Verification

As shown in Table 3, the predicted values obtained from Equation (7) reveal good agreement with the experimental values. The average ratio of the predicted values to the experimental tensile values is 0.992, and the standard deviation is 0.005. Table 3 shows that the moisture damage factors of the steam-curing, *λ*, reflects the effects of the thickness of the protective layer and the diameter of GFRP bars on the tensile strength of GFRP bars embedded in the steam-curing concrete beams and decreases with the increase of the thickness of the protective layer and the increase of the diameter.

The above analysis shows that the semi-reliable Probabilistic Damage Assessment and prediction model based on the moisture absorption model of multiple related factors is effective and can capture the damage response of GFRP rods embedded in steam cured concrete beams.

## 5. Conclusions

At present, GFRP bar as a structural material has been widely used in steam cured concrete beams. GFRP bar will be damaged due to a series of irreversible hygroscopic chemical reactions under humid and hot curing environmental conditions. It is of excellent engineering and academic significance to study the damage of GFRP bars caused by various factors related to the moisture absorption model. The essential factor of the damage could be precisely analyzed by studying the multiple factors related to the moisture absorption performance of GFRP bars. In addition, some errors, certain assumptions, and uncertainties will be prevented in the prediction equations of Fick’s law. In order to establish the semi-reliable probability damage model of GFRP bars in steam cured concrete beams, the following measures could be taken: improvement of the theoretical research on the design of steam cured GFRP reinforced concrete members, providing some theoretical guidance for the design of steam cured GFRP reinforced concrete members, and solving the problems such as the design life of steam cured concrete members.

(1) In this paper, the change of microstructure morphology shows that the resin damage of GFRP bars decreases with increasing diameter. The maximum moisture absorption rates of GFRP bars with a diameter of 10, 16, 19, and 22 mm embedded in steam-curing concrete beams, respectively, were 0.652%, 0.52%, 0.318%, and 0.25%, which respectively were 0.57, 0.67, 0.59, and 0.76 times that of the bare steel. It means the deterioration of moisture absorption performance would decrease with increasing diameter.
(2) It was revealed that the influence of the steam-curing concrete environment on the moisture absorption rate of GFRP bars was not consistent with the standard-curing concrete environment. The maximum moisture absorption rate of GFRP bare bars reached 8 times as much as the GFRP bars embedded in standard-curing concrete. The maximum moisture absorption rate and the slope of the linear stage of GFRP bars embedded in steam-curing concrete beams with the steam-curing temperature of 60 °C respectively were 2.72 times and 3 times the GFRP bars embedded in standard-curing concrete. The impact of the steam-curing concrete environment on the moisture absorption rate of GFRP bars was the most obvious compared to the bare and the standard concrete environment, and the degree of influence increased with an increase of the curing temperature.
(3) The maximum moisture absorption rate and the slope of the linear stage decrease with increasing thickness of the protective layer. The maximum moisture absorption rates of GFRP bars embedded in steam-curing concrete beams with the protective layer of 15, 20, 25, and 35 mm were approximately 0.81%, 0.69%, and 0.628%, respectively. The slopes of the linear stage were 2.1, 1.6, 1.4, and 1.3, respectively. This means that with the increase of the thickness of the protective layer, the deterioration of the moisture absorption performance will decrease.
(4) One of the more significant findings to emerge from this study was that the moisture absorption performance of GFRP bars embedded in steam-curing concrete beams was influenced by multi factors, including the curing temperature, the diameter, and the thickness of the protective layer. The diffusion coefficient of GFRP bars steam-curing concrete beams increased with increasing temperature and diameter and decreased with increasing the thickness of the protective layer. Multivariate regression analysis was specialized to establish the multiple factors related moisture absorption diffusion model, which was found to be in close agreement with the experiment values.
(5) Based on the multiple factors related moisture absorption model, the semi-reliability probability damage assessment was proposed in this paper by introducing the moisture damage factors, the random variable, and the error term. The average ratio and the standard deviation of the predicted values to the experimental tensile values were 0.992 and 0.005, respectively. It shows that the semi-reliability probability damage assessment is effective and capable of capturing the damage response of GFRP bars embedded in steam-curing concrete beams.

## Figures and Tables

**Table 1:** Test Specimens.

Environment	Diameter (mm)	Curing Temperature	Thickness of Protective Layer (mm)	Moisture Absorption Test (mm)	Tensile Strength Test (mm)	Working Condition Abbreviation
GFRP bare bars	10	-	-	200	1100	RE-10
	16	-	-			RE-16
	19	-	-			RE-19
	22	-	-			RE-22
GFRP bars embedded in concrete	10	20 °C	25	200 × 110 × 80	1100 × 110 × 80	G-Sta
		60 °C	15			G-Ste1-1
			20			G-Ste1-2
			25			G-Ste1-3
			35			G-Ste1-4
		80 °C	25			G-Ste80
	16	60 °C	25			G-Ste2
	19	60 °C	25			G-Ste3
	22	60 °C	25			G-Ste4

**Table 2:** Diffusion coefficient of GFRP bars.

Working Condition Abbreviation	Diameter (mm)	Curing Temperature	Thickness of Protective Layer (mm)	$\sqrt{t_{1}}$ (hr^0.5^)	*M_t_*_1_	$\sqrt{t_{2}}({\mathrm{hr}}^{0.5})$	*M_t_*_2_	*M_m_*	Diffusion Coefficient *D* (mm^2^/s)
RE-10	10	-	-	$\sqrt{24}$	0.18	$\sqrt{888}$	0.72	1.08	2.2 × 10^−6^
RE-16	16	-	-		0.12		0.50	0.72	6.3 × 10^−6^
RE-19	19	-	-		0.07		0.41	0.50	14.7 × 10^−6^
RE-22	22	-	-		0.02		0.28	0.31	22.3 × 10^−6^
G-Sta	10	20 °C	25		0.05		0.14	0.17	2.5 × 10^−6^
G-Ste1-1		60 °C	15		0.24		0.75	0.79	3.7 × 10^−6^
G-Ste1-2			20		0.18		0.59	0.68	3.2 × 10^−6^
G-Ste1-3			25		0.16		0.53	0.64	2.9 × 10^−6^
G-Ste1-4			35		0.13		0.46	0.61	2.6 × 10^−6^
G-Ste80		80 °C	25		0.21		0.65	0.74	3.1 × 10^−6^
G-Ste2	16	60 °C	25		0.06		0.34	0.47	7.3 × 10^−6^
G-Ste3	19	60 °C	25		0.02		0.24	0.31	16.1 × 10^−6^
G-Ste4	22	60 °C	25		0.01		0.22	0.24	24.3 × 10^−6^

**Table 3:** Comparison of experimental and predicted values.

Working Condition Abbreviation	Diameter (mm)	Thickness of Protective Cover (mm)	Curing Time *t* (10^6^ s)	Moisture Damage Factors		Tensile Strength of GFRP Bars (MPa)		
				*λ* (10^−12^)	*γ*	Experimental Values	Predicted Values	Predicted Values/Experimental Values
G-Ste1-1	10	15	2.4192	1.50	1.69	1183	1180.071	0.998
G-Ste1-2	10	20	2.4192	1.41	1.69	1200	1187.215	0.989
G-Ste1-3	10	25	2.4192	1.35	1.69	1208	1192.462	0.987
G-Ste1-4	10	35	2.4192	1.25	1.69	1212	1199.917	0.990
G-Ste2	16	25	2.4192	0.89	1.69	870	869.746	1.000
G-Ste3	19	25	2.4192	0.82	1.69	745	740.716	0.994
G-Ste4	22	25	2.4192	0.79	1.69	702	694.346	0.989
Average value								0.992
Standard deviation								0.005

## Data Availability

All data generated or analyzed during this study are included in this published article.
